# Tetrathiafulvalene-based azine ligands for anion and metal cation coordination

**DOI:** 10.3762/bjoc.11.149

**Published:** 2015-08-07

**Authors:** Awatef Ayadi, Aziz El Alamy, Olivier Alévêque, Magali Allain, Nabil Zouari, Mohammed Bouachrine, Abdelkrim El-Ghayoury

**Affiliations:** 1 Laboratoire MOLTECH Anjou, Université d’Angers, UFR Sciences, UMR 6200, CNRS, Bât. K, 2 Bd. Lavoisier, 49045 Angers Cedex, France; 2 Laboratoire de Physico-chimie de l’état solide, Université de Sfax, Route de Soukra; Km 4; BP: 802, 3038, Sfax, Tunisia; 3 MEM, High School of Technology (ESTM), University, Moulay Ismail, Meknès, Morocco

**Keywords:** azine ligand, fluoride sensing, rhenium, tetrathiafulvalene, X-ray

## Abstract

The synthesis and full characterization of two tetrathiafulvalene-appended azine ligands, namely 2-([2,2’-bi(1,3-dithiolylidene)]-4-yl)-6-((2,4-dinitrophenyl)hydrazono)methyl)pyridine (**L1**) and 5-([2,2’-bi(1,3-dithiolylidene)]-4-yl)-2-((2,4-dinitrophenyl)hydrazono)methyl)pyridine (**L2**) are described. The crystal structure of ligand **L1** indicates that the ligand is completely planar with the presence of a strong intramolecular N3–H3···O1 hydrogen bonding. Titration experiments with inorganic anions showed that both ligands are suitable candidates for the sensing of fluoride anions. Ligand **L2** was reacted with a Re(I) cation to yield the corresponding rhenium tricarbonyl complex **3**. In the crystal structure of the newly prepared electroactive rhenium complex the TTF is neutral and the rhenium cation is hexacoordinated. The electrochemical behavior of the three compounds indicates that they are promising for the construction of crystalline radical cation salts.

## Introduction

Tetrathiafulvalene (TTF) is known to have excellent electron-donating properties resulting in stable radical cation (TTF^•+^) and dication (TTF^2+^) species from two sequential and reversible oxidation processes. The huge interest in the synthesis of TTF and its very numerous derivatives [1] has been initiated by the high electrical conductivity discovered in a chloride salt of TTF [2] and metallic behavior in the charge-transfer complex with 7,7,8,8-tetracyano-*p*-quinodimethane (TCNQ) [3]. These systems have played a major role for the preparation of molecular materials designed for various applications. They have been, for example, used as electron donor molecules to prepare electrically (super)conducting crystalline materials [4–7], as solar energy systems [8–9] or even as donor moieties in nonlinear optical (NLO) materials [10–11]. In the last decades one of the biggest challenges in materials science is devoted to the preparation of multifunctional molecular materials that can potentially exhibit, in solution and/or in solid state, synergy or coexistence between two or more different physical properties. In order to address this issue, many efforts have been devoted to the association of a binding or coordinating unit to the redox-active TTF moiety. This strategy has led for example, in solid state, to the preparation of electroactive metal complexes that combine magnetic and electrical properties [12–17]. In solution, TTF-based redox-responsive receptors for neutral and/or charged guest sensing applications have been prepared [18–22]. On this ground, chemosensors capable of recognizing anionic and/or cationic species constitute an important area of increasing research in supramolecular chemistry, considering the ubiquitous properties of both anions and metal cations [23–27]. In fact, anions are involved in a large number of biological and chemical processes [28–31]. Note that fluoride is of particular interest among the other inorganic anions because of its both beneficial (e.g., preventing dental caries and treatment of osteoporosis) and detrimental (e.g., fluorisis) effects on human health [32–34]. Many TTF derivatives have been used to coordinate or to bind separately metal cations [35] or inorganic anions [36], however only few examples were used for both inorganic anions and metal cations coordination [37].

**Scheme 1:**
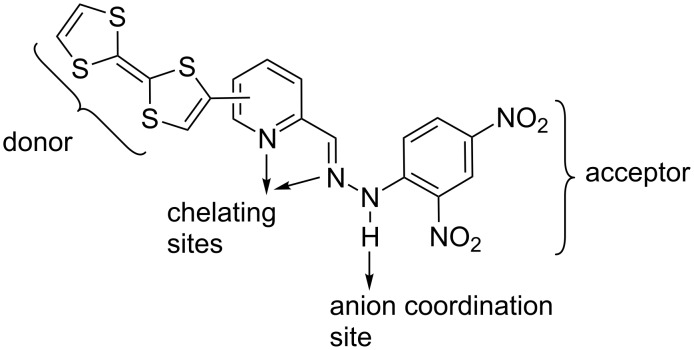
Multifunctional TTF-appended azine ligands.

Herein we report the synthesis and electronic properties of two new multifunctional TTF-based azine ligands that integrate distinctive functional groups as depicted in Scheme 1 and thus capable of coordinating both metal cations as well as inorganic anions. The sensing studies for inorganic anions are discussed. Metal cation complexation studies of the new ligands afforded the formation and the structural characterization of a neutral rhenium complex.

## Results and Discussion

### Synthesis of the ligands **L1** and **L2**

**Scheme 2:**
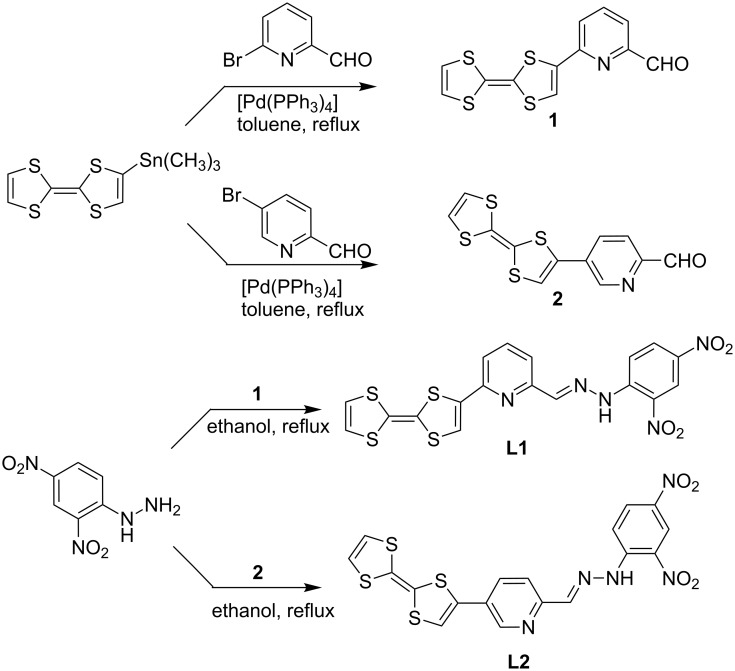
Synthetic scheme for TTF-based azine ligands **L1** and **L2**.

The protocol followed for the synthesis of the new azine ligands **L1** and **L2** is summarized in Scheme 2. The reaction of 6-bromo-2-pyridinecarboxaldehyde or 5-bromo-2-pyridinecarboxaldehyde with one equivalent of TTF-SnMe_3_ under the Stille coupling conditions, using [Pd(PPh_3_)_4_] as catalyst in toluene, afforded the intermediates 6-([2,2’-bi(1,3-dithiolylidene)]-4-yl)picolinaldehyde (**1**, previously described in reference [38]) and 5-([2,2’-bi(1,3-dithiolylidene)]-4-yl)picolinaldehyde (**2**) in good yields (60% and 65%, respectively), after chromatographic work-up. Condensation of pyridinealdehyde-functionalized TTF **1** or **2** with 2,4-dinitrophenylhydrazine, in refluxing ethanol, afforded the desired ligands **L1** and **L2** in 75% and 63% isolated yields, respectively. The structures of the new ligands were characterized by ^1^H and ^13^C NMR, UV–visible and IR spectroscopy, high resolution mass spectrometry and elemental analysis.

### Crystal structure description

Details about data collection and structure refinement are given in Table 1. Crystallographic data for the structural analysis have been deposited within the Cambridge Crystallographic Data Centre, CCDC 1055120 (ligand **L1**) and CCDC 1055119 (complex **3**).

**Table 1:** Crystal data and structure refinement for ligand **L1** and complex **3**.

compound	Ligand **L1**	Complex **3**

Empirical formula	C_78_H_62_N_20_O_19_S_19_	C_42_H_24_Cl_2_N_10_O_15_Re_2_S_8_
fw	2192.62	1608.49
*T* (K)	293(2)	180.0(1)
wavelength (Å)	0.71073	1.54184
cryst syst	Orthorhombic	Triclinic
space group	Pbca	*P*1
*a* (Å)	7.353(3)	8.591(1)
*b* (Å)	30.35(1)	11.501(1)
*c* (Å)	20.894(6)	15.567(2)
α (deg)	90	99.494(8)
*β* (deg)	90	100.97(1)
*γ* (deg)	90	94.926(8)
*V* (Å^3^)	4663(3)	1478.3(3)
*Z*	*2*	1
*D*_c_ (g cm^−3^)	1.562	1.807
abs coeff (mm^−1^)	0.517	11.955
*F*(000)	2252	778
cryst size (mm^3^)	0.26 x 0.04 x 0.02	0.2061 x 0.0406 x 0.0194
θ range for data collection (deg)	3.01–24.98	2.94–72.85
Tmin/Tmax	0.867 / 0.990	0.656 / 1.000
reflns collected	32107	8638
indep reflns	4078	5473
completeness (%)	99.5	96.8
R(int)	0.1083	0.1495
refinement method	full-matrix least squares on *F *^2^	full-matrix least squares on *F *^2^
Data with [ *I* > 2*σ*(*I*)] /restraints/param	1974/ 0 / 280	3616 / 7 / 361
GOF on *F *^2^	1.093	1.041
final *R* indices [*I* > 2*σ*(*I*)]	R1 = 0.0947, wR2 = 0.1705	R1 = 0.0987, wR2 = 0.2569
*R* indices (all data)	R1 = 0.2089, wR2 = 0.2046	R1 = 0.1458, wR2 = 0.2810
largest diff. peak and hole (e Å^−3^)	0.255 and −0.310	2.290 and −2.053
X-ray wavelength radiation	Mo K_α_	Cu K_α_

**Figure 1:**
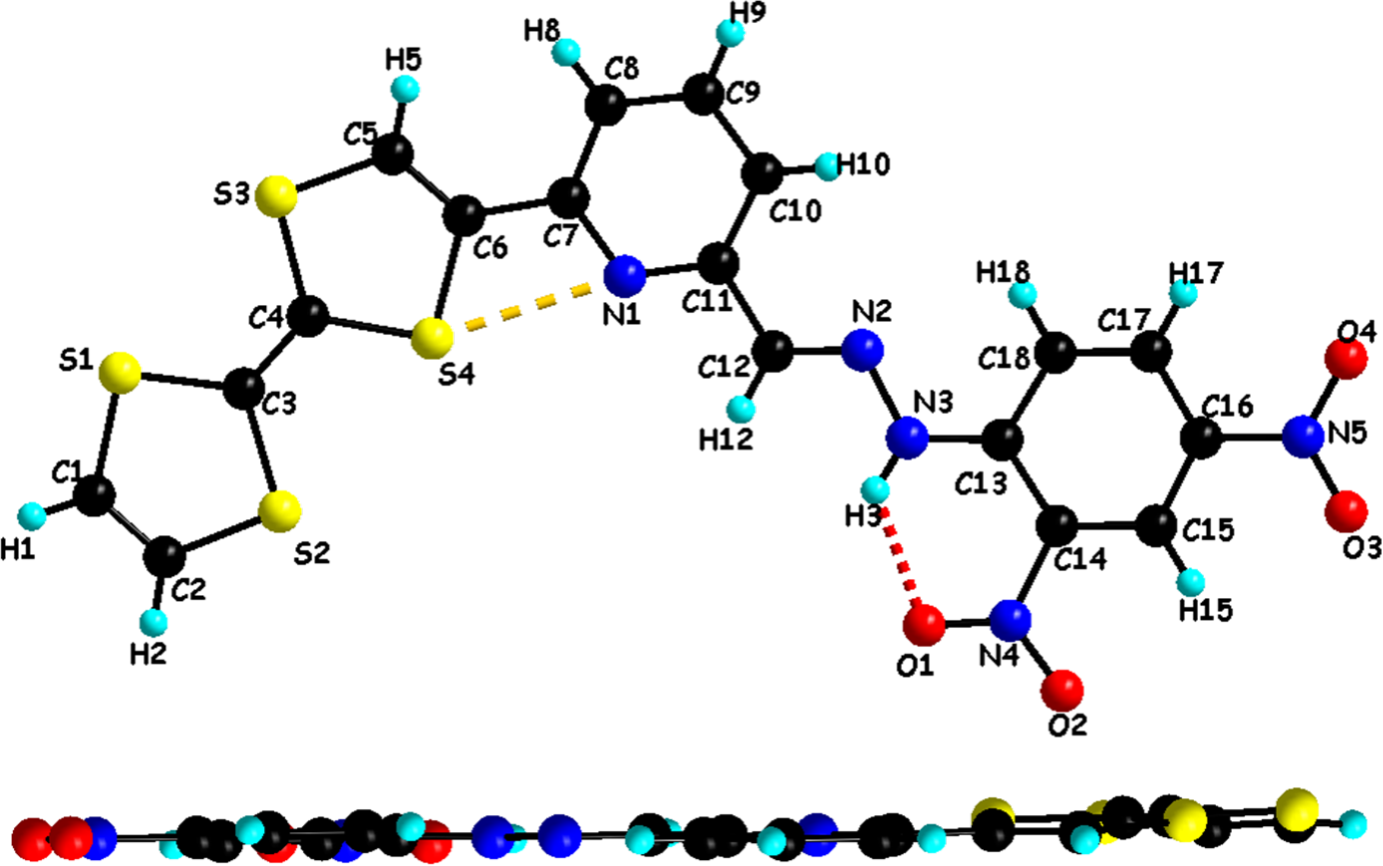
Crystal structure of ligand **L1** with atom numbering scheme (top) and a side view of the molecule (bottom).

Suitable single crystals for X-ray analysis have been grown for ligand **L1** upon recrystallization from DMSO solution. Ligand **L1** crystallizes as dark plates in the orthorhombic system, space group Pbca and selected bond lengths and angles are depicted in Table 2. As it can be seen in Figure 1, ligand **L1** is completely planar. The dihedral angle between the dinitrophenylhydrazone and pyridine planes is 2.67 (2)° which is slightly lower than the dihedral angle observed in the case of the pyridine-2-carbaldehyde 2,4-dinitrophenylhydrazone that is 3.88 (8)° [39]. The molecular conformation of the ligand is stabilized by a strong intramolecular N3–H3···O1 (2.015 (6) Å) hydrogen bond making the dinitrophenyl ring coplanar with the hydrazone unit and also by an intramolecular short contact S4···N1 (2.824(6) Å) that makes the TTF unit and the pyridine ring coplanar.

**Table 2:** Selected bond lengths (Å) and angles (°) in **L1**.

Bond length (Å)			

C1–S1	1.749(8)	C6–C7	1.487(9)
C1–C2	1.310(9)	C7–N1	1.361(8)
C2–S2	1.745(7)	N1–C11	1.324(8)
C3–S2	1.789(7)	C11–C12	1.463(9)
C3–S1	1.748(8)	C12–N2	1.256(8)
C3–C4	1.349(9)	N2–N3	1.383(7)
C4–S3	1.741(7)	N3–C13	1.340(8)
C4–S4	1.779(7)	C13–C14	1.431(9)
S4–C6	1.777(6)	C14–N4	1.474(9)
C6–C5	1.337(8)	N4–O1	1.217(8)

Angle values (°)			

N1–C11–C10	123.7(7)	N2–N3–C13	119.0(6)
C12–N2–N3	117.2(6)	C18–C13–N3	120.3(6)
C11–C12–N2	121.9(7)	C13–C14–N4	120.9(7)

**Figure 2:**
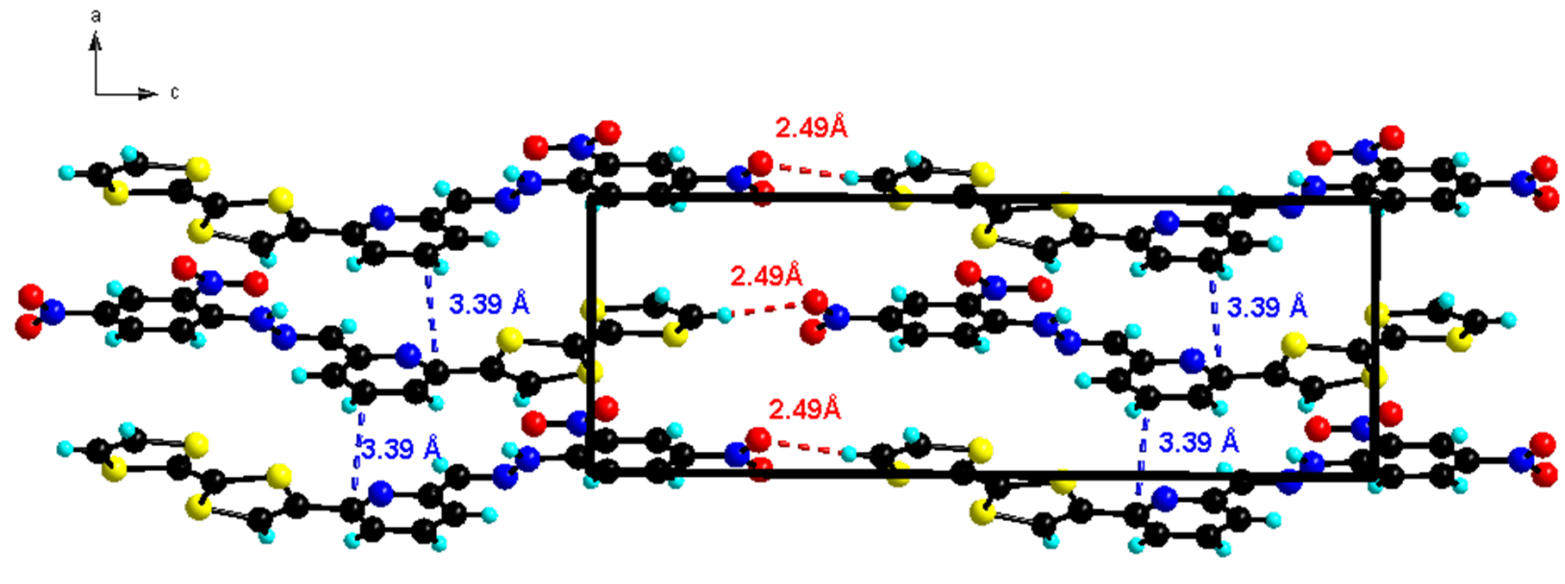
Partial crystal packing of ligand **L1** with formation of head to tail dimers that stack along *a*-axis forming columns that are connected through hydrogen bonding along *c*-axis.

In the solid state, the packing arrangement in **L1** is characterized by the self-assembly between the TTF donor unit and the dinitrophenyl acceptor unit forming head-to-tail dimers. Moreover, the plane-to-plane distance between the donor and acceptor moieties is *d* = 3.39 Å, showing an evident overlap that is comparable to the reported intermolecular charge-transfer complexes [35]. This overlap develops along *a*-axis forming infinite columns (Figure 2). These columns are linked together along *c*-axis through hydrogen bonding occurring between TTF-C-H and NO_2_ (TTF-C–H1···O3-NO 2.493(6) Å).

**Figure 3:**
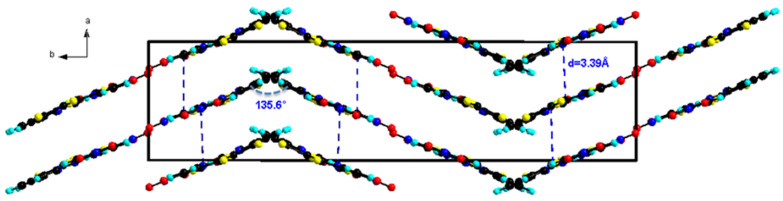
Packing diagram of **L1** showing the orientation of the columns of head to tail dimers.

In the *b*-direction, the columns of stacked head to tail molecules are connected laterally through S···O heteroatom contacts (*d* = 3.16 Å, Figure S1, Supporting Information File 1) resulting in alternating stacks with a “zig zag” like manner with an angle of rotation of 135.6° (Figure 3).

### UV–visible absorption spectroscopy

**Figure 4:**
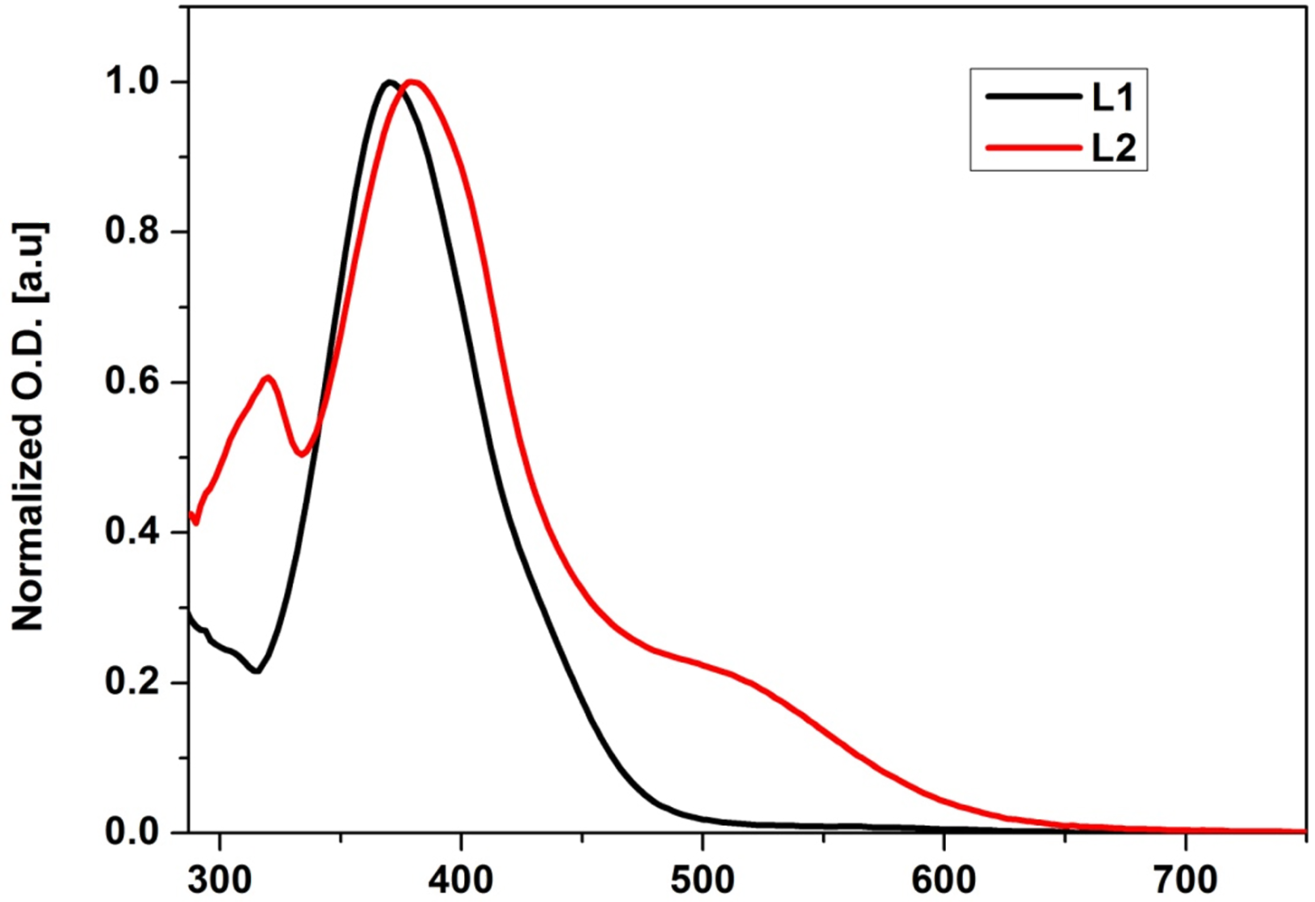
UV–visible absorption spectra of ligands **L1** and **L2** (*c* 2.5 × 10^−5^ M in (dichloromethane/acetonitrile, 9:1, v/v)), room temperature.

The UV–visible absorption spectra of the ligands **L1** and **L2** were recorded in a mixture of dichloromethane/acetonitrile solution (9/1, v/v, C = 2 × 10^−5^ M) at room temperature (Figure 4). The two ligands exhibit strong electronic absorption bands between λ = 300 nm and 450 nm which are assigned to the π→π* and n→π* absorption bands resulting from the different units of the two ligands (TTF moiety, pyridyl ring and the dinitrophenylhydrazone group). As compared to ligand **L1**, **L2** exhibits an additional absorption band around λ = 516 nm which is attributed to an intramolecular charge transfer (ICT) excitation from the TTF donor moiety to the dinitrophenylhydrazone accepting group. These results from a strong π-electronic delocalization that occurs in ligand **L2** leading to a resonance structure that involves the C=N hydrazone bond as it can be seen in (Figure S2, Supporting Information File 1).

### Theoretical calculations

**Figure 5:**
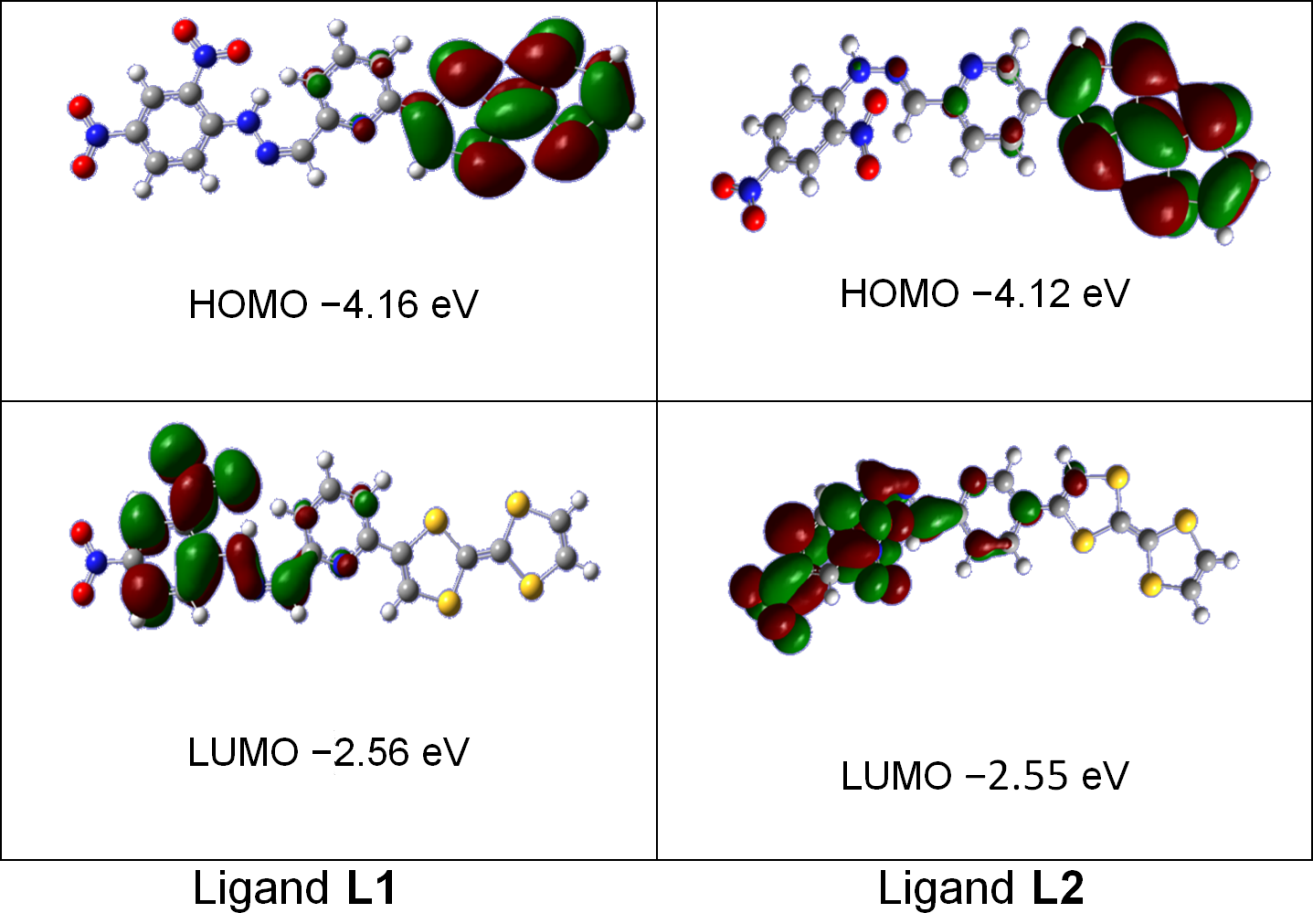
HOMO–LUMO Frontier orbitals representation for ligands **L1** and **L2**.

Theoretical calculations based on density functional theory (DFT) methods have been performed with the Gaussian 09 program [40]. Becke’s three-parameter gradient-corrected functional (B3LYP) with 6-31G (d) basis in vacuum was used for full geometry optimization of the two ligands. The resulting frontier molecular orbitals (Figure 5) for ligands **L1** and **L2** indicate that the electron density of the highest occupied molecular (HOMO) orbitals develop exclusively on the TTF fragment. The LUMO orbital for ligand **L1** is essentially distributed on the nitrophenylhydrazino group with a small participation of the pyridyl ring, while for ligand **L2** it is distributed on the π-extended system with a small participation of the external ethylenic atom of the TTF moiety which is confirming the good electronic conjugation in this ligand. The same behavior is observed for the TTF pyridine carboxaldehyde precursors **1** and **2** (Figure S3, Supporting Information File 1).

### Cyclic voltammetry

**Figure 6:**
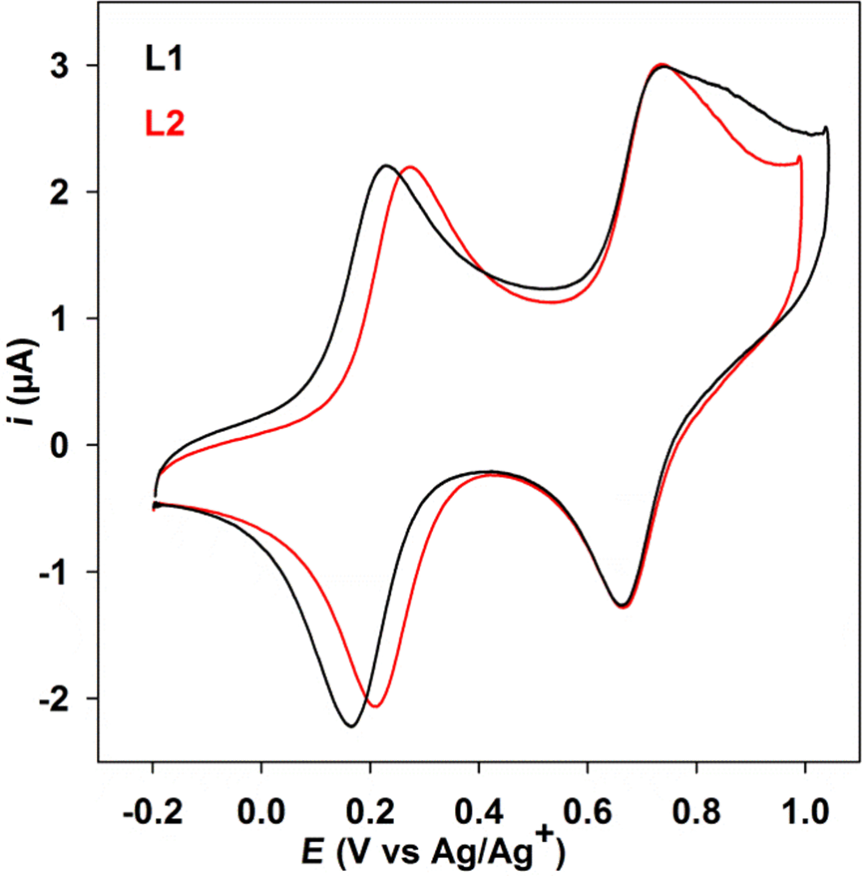
Cyclic voltammograms of ligands **L1** and **L2** (2 × 10^−5^ M) in CH_2_Cl_2_/CH_3_CN (9:1, v/v) at 100 mV·s^−1^ on a glassy carbon electrode with *n-*Bu_4_NPF_6_ (0.1 M).

The electrochemical behavior of the electroactive precursors **1** and **2** as well as of ligands **L1** and **L2** was investigated by cyclic voltammetry (Figure 6 and Table 3). The measurements in the case of precursors **1** and **2** show two reversible oxidations at *E*_1ox_ = +0.26 V, *E*_2ox_ = +0.75 V and *E*_1ox_ = +0.32 V, *E*_2ox_ = +0.77 V vs Ag/Ag^+^, respectively, that are anodically shifted when compared to the ones of the free TTF because of the presence of the electron deficient pyridinecarboxaldehyde moiety. In addition, *E*_1ox_ of **2** is anodically shifted when compared with *E*_1ox_ of **1**, indicating a strong π-electron conjugation in precursor **2**. As for **1** and **2**, ligands **L1** and **L2** show two reversible oxidations at *E*_1ox_ = +0.20 V, *E*_2ox_ = +0.70 V and *E*_1ox_ = +0.25 V, *E*_2ox_ = +0.70 V vs Ag/Ag^+^, respectively) that are cathodically shifted when compared to the ones of **1** and **2** indicating that the pyridine-hydrazone group is less electron deficient than the corresponding pyridinecarboxaldehyde. In addition, *E*_1ox_ of **L2** is also anodically shifted when compared with *E*_1ox_ of **L1** because of the strong π-electron conjugation in ligand **L2** and this is in agreement with the bathochromic shift observed for **L2** in the UV–visible absorption spectra.

**Table 3:** Apparent redox potentials (V) of molecular compounds **1, 2, L1** and **L2** reported vs Ag/Ag^+^ (0.01 M) in 0.1 M TBAPF_6_ in CH_2_Cl_2_/CH_3_CN 3:1 on glassy carbon electrode at 100 mV·s^−1^.

compound	*E*_ox1_	*E*_ox2_

**1**	0.26	0.75
**2**	0.32	0.77
**L1**	0.20	0.70
**L2**	0.25	0.70

### Sensing properties of the azine ligands for anions

**Figure 7:**
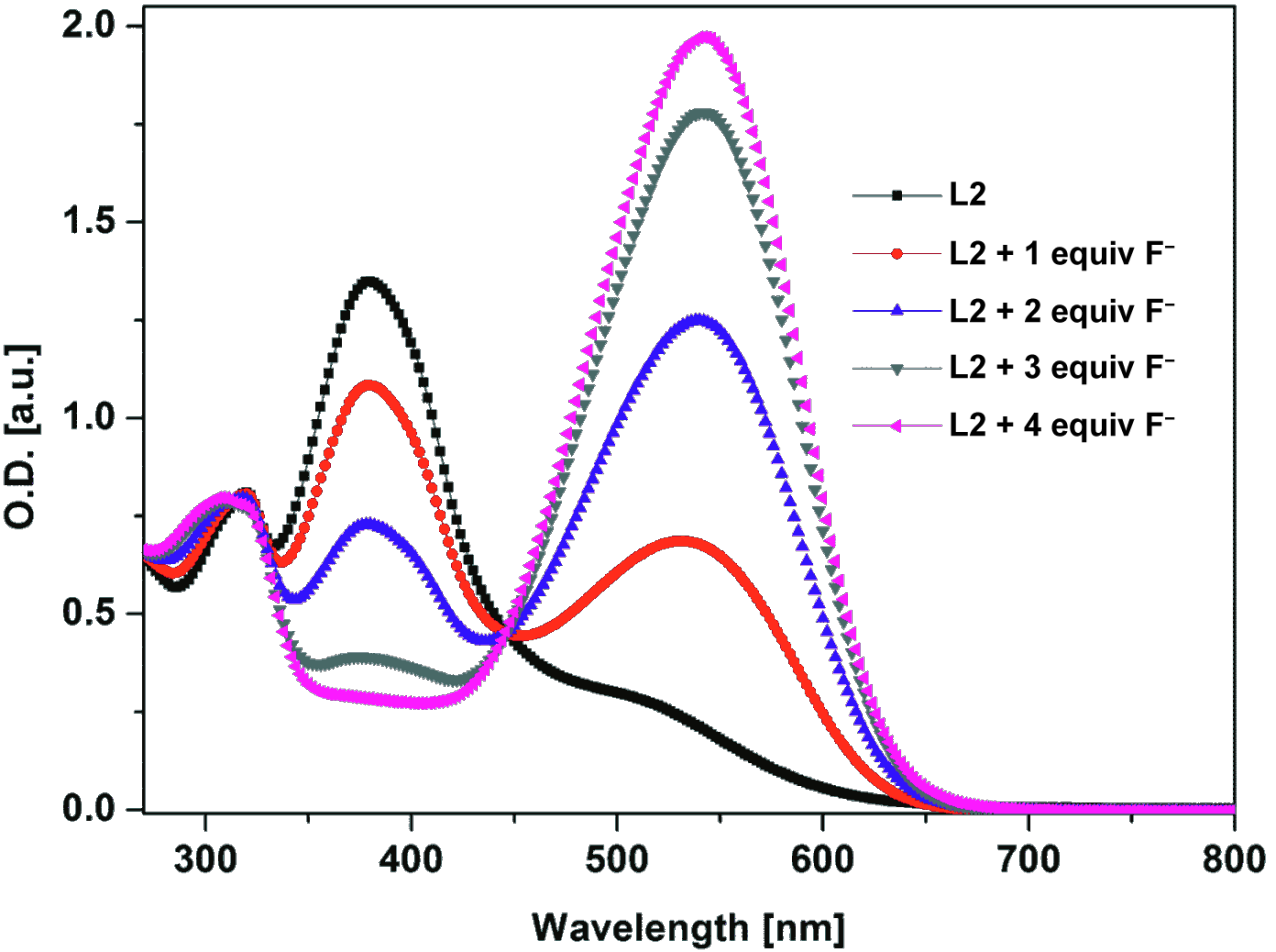
UV–visible spectral changes of ligand **L2** (2 × 10^−5^ M in CH_2_Cl_2_/CH_3_CN, 9/1) upon addition of TBAF.

It is known that phenylhydrazone groups are able to act as optical sensors particularly for fluoride anions [41–45]. Thus, the colorimetric sensing abilities of the two ligands **L1** and **L2** were investigated by adding various anions such as hydrogensulfate, acetate, iodine and fluoride (used as tetrabutylammonium salts) in a mixture of dichloromethane/acetonitrile (9:1, v/v). Addition of increasing amounts of F^−^ causes a dramatic change in color from yellow to violet that can be observed by the naked eye (Figure S4 in Supporting Information File 1), which is accompanied by the formation of a new broad absorption band centered at about 510 nm in the case of ligand **L1**. In the case of ligand **L2**, addition of F^−^ (Figure 7) causes also a dramatic change in color from light orange to violet that can be observed by the naked eye (Figure S5 in Supporting Information File 1), that is accompanied by a decrease of the intense absorption band centered at about 380 nm and the increase of the ICT absorption band centered around 540 nm. This change is likely due to the deprotonation of the hydrazone nitrogen which causes an enhancement of charge transfer from the TTF unit and the deprotonated nitrogen to the electron poor 2,4-dinitrophenyl moiety [36]. A remarkable feature is the occurrence of a quite well defined isosbestic point at 420 nm and 447 nm for **L1** and **L2**, respectively, indicating that **L1** or **L2** coexist with only one species upon addition of TBAF. Note that upon addition of other inorganic anions such as bromide, chloride or hydrogensulfate we have observed a negligible absorption changes while in the case of acetate anion a moderate absorption changes are obtained (Figures S6 and S7 in Supporting Information File 1) [46].

Treatment of an electrolytic solution of ligand **L1** or **L2** with an increasing amount of fluoride anion (tetrabutylammonium fluoride trihydrate in a CH_2_Cl_2_/CH_3_CN mixture) involve the presence, as previously seen for fluoride anion sensing [47], and mainly on the first cycle, of the pre-wave superimposed on the wave of oxidation of the ligands. We clearly see on the second cycle a negligible change of the oxidation potential of the ligand which is very likely because of the large distance between the TTF and the fluoride coordinating unit (Figure S8 in Supporting Information File 1).

**Figure 8:**
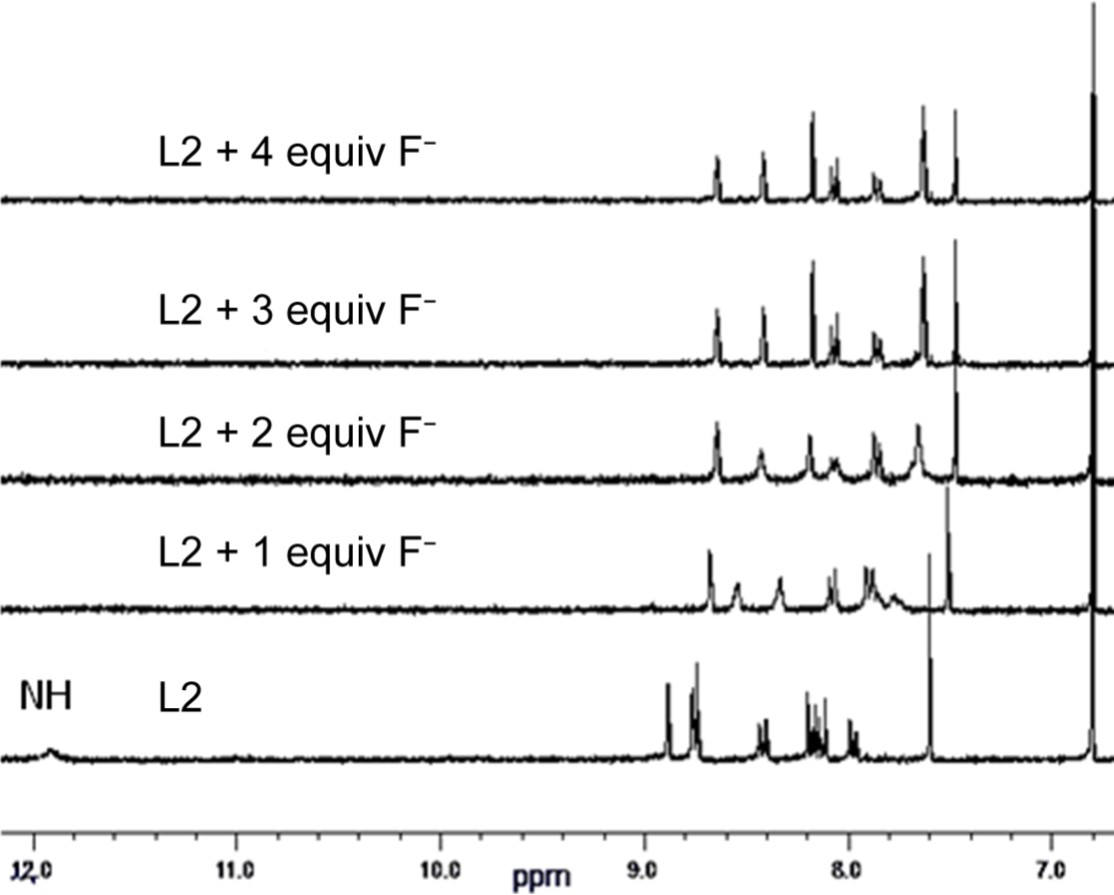
^1^H NMR spectra of ligand **L2** (4·10^−3^ M in DMSO-*d*_6_) upon addition of successive aliquots of TBAF (DMSO-*d*_6_).

In order to get further supports to the observed optical sensing and to get deeper insights into the interactions between **L1** or **L2** and fluoride, ^1^H NMR titration experiments were performed in DMSO-*d*_6_ (Figure 8 and Figure S9 in Supporting Information File 1). The measurements indicate that the N–H peak disappears after addition of one equivalent of TBAF while the other aromatic proton resonances of **L1** or **L2** exhibit an upfield shift. These results tend to be consistent with the deprotonation of the N–H group and the delocalization of the negative charge over the π-conjugated system as previously observed TTF dinitrophenylhydrazone [36]. Note that there is no change in the ^1^H NMR spectrum observed for other anions.

### Synthesis and crystal structure of a neutral rhenium complex

**Figure 9:**
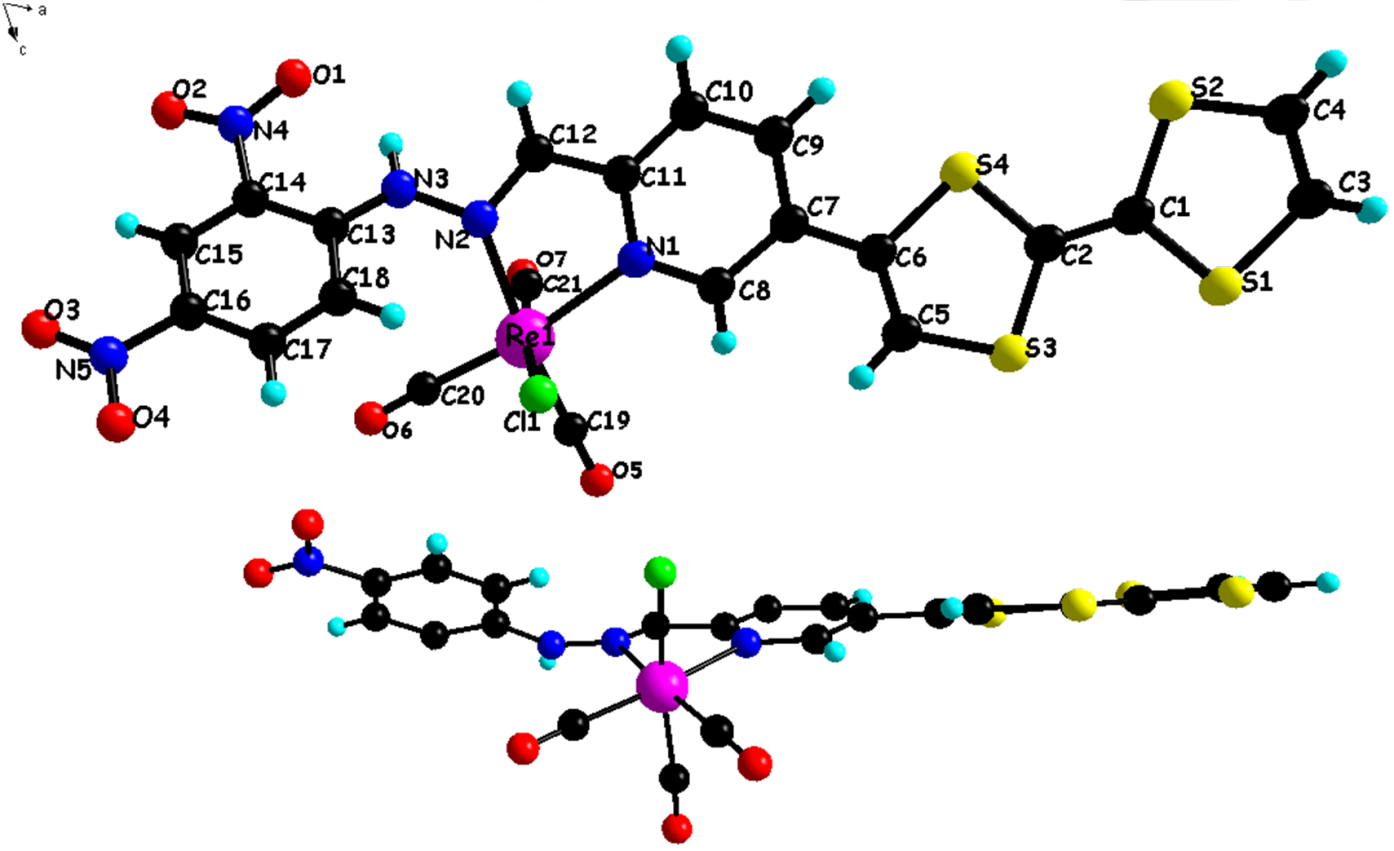
Crystal structure of complex **3** with atom numbering scheme (top) and a side view of the molecule (bottom). Water molecules are omitted for clarity.

Few metal complexes based on ruthenium cations have been previously prepared with dinitrophenylazine type ligands [48–49]. These reports indicate that the pyridinedinitrophenylazine type ligands are good candidates for the formation of metal complexes. We have therefore investigated the complexation of **L1** and **L2** with various metal cations and we succeeded in the crystallization of a neutral rhenium metal complex with ligand **L2**. Thus, the equimolar reaction between **L2** and the [Re(CO)_5_Cl] precursor performed in refluxing toluene, under no light and inert atmosphere, afforded a mononuclear neutral complex **3** described as [Re**L2**(CO)_3_Cl]·0.5H_2_O as a dark precipitate [50]. Single crystals of **3** were obtained by recrystallization from acetone/hexane solution. Details about data collection and structure refinement are given in Table 1. As expected, the resulting metal complex **3** is composed of one ligand **L2** coordinated to Re(CO)_3_Cl fragment through two nitrogen atoms of the pyridine and the C=N hydrazone group (Figure 9). Upon complexation, the ligand acquires a *cis-*conformation of the hydrazinopyridine moiety in contrast to the *trans*-conformation observed for free ligand **L1** (see Figure 1 and Figure 9). Within the complex, the rhenium center is surrounded by the bidentate chelating **L2** ligand, three carbonyl ligands arranged in a facial fashion, and a chlorine atom and its coordination sphere presents the expected, although slightly distorted, octahedral geometry. The angle formed by the rhenium center and N atoms equals to 73.6(5)° which is smaller than the angle of 90° adopted in an ideal octahedron. In addition, in the complex the C–Re–C angles identified as C19–Re1–C20, C19–Re–C21, C20–Re–C21 are 87.7°, 91.7° and 90.9°, respectively, which are close to 90° indicating that CO ligands are almost linearly coordinated to the rhenium(I) cation. The length of the two Re–N bonds are (N1–Re1 2.20(1) Å) and N2–Re1 2.18(1) Å, and the formal double bond character C=N is maintained (C12–N2 1.32(2) Å). All three Re–CO bond lengths are very close, and the Re–C–O angles present minor deviations from linear structure, values ranging from 165(2)° to 177(2)° (Table 4).

**Table 4:** Selected bond lengths (Å) and angles (°) in complex **3**.

Bond length (Å)			

N1–Re1	2.20(1)	C20–Re1	1.96(2)
N2–Re1	2.18(1)	C19–Re1	1.89(2)
Cl1–Re1	2.39(8)	C11–N1	1.35(2)
C21–Re1	2.19(1)	C11–C12	1.43(2)

Angle values (°)			

C19–Re1–C20	87.7(9)	N2–Re1–N1	73.6(5)
C19–Re1–C21	91.7(7)	C21–Re1–Cl1	172.7(4)
C20–Re1–C21	90.9(7)	C19–Re1–Cl1	89.7(6)
C19–Re1–N2	174.2(6)	C20–Re1–Cl1	96.3(7)
C20–Re1–N2	97.4(8)	N1–Re1–Cl1	86.1(4)
C19–Re1–N1	101.1(7)	N2–Re1–Cl1	92.4(4)
C20–Re1–N1	170.8(8)	O5–C19–Re1	177(2)
C21–Re1–N2	85.6(5)	O6–C20–Re1	177(2)
		O7–C21–Re1	165(2)

**Figure 10:**
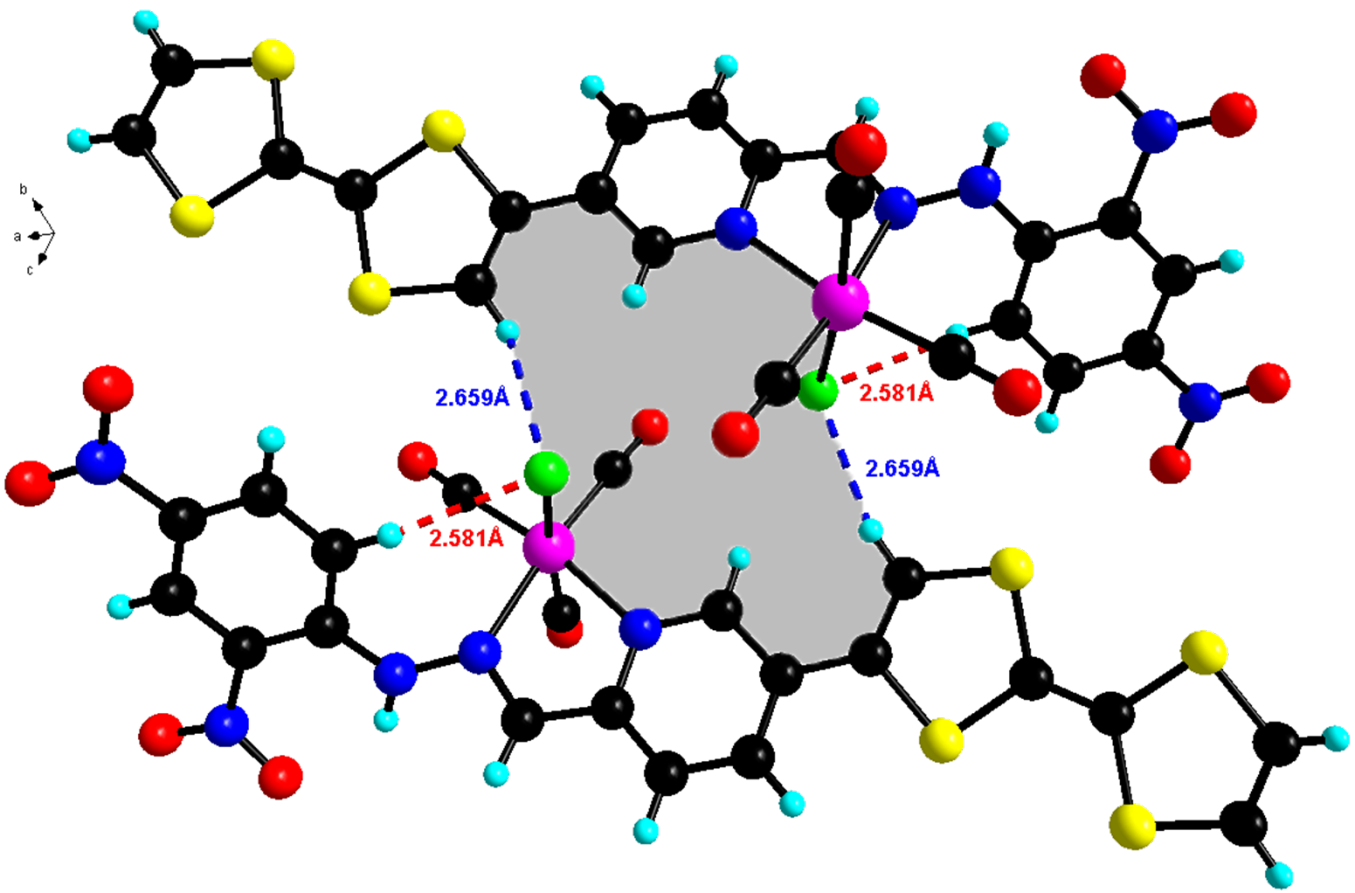
Pattern of intramolecular and intermolecular contacts in **3**. Two molecules are linked by pairs of strong TTF-C–H···Cl hydrogen bonds forming R_2_^2^(16) cyclic motifs (in grey filling).

In the crystal structure, the chlorine atom coordinated to rhenium is involved in an intramolecular C–H···Cl hydrogen bonding with the hydrogen from the pyridyl ring with a distance of 2.581(6) Å. In addition, it is involved in an intermolecular hydrogen bonding with a neighboring molecule by a strong TTF-C–H···Cl bond (2.659(6) Å) resulting in the formation of dimers that are formed with a R_2_^2^(16) cyclic motif (grey filling in Figure 10) as it was previously observed within a catechol-appended TTF derivative [51].

**Figure 11:**
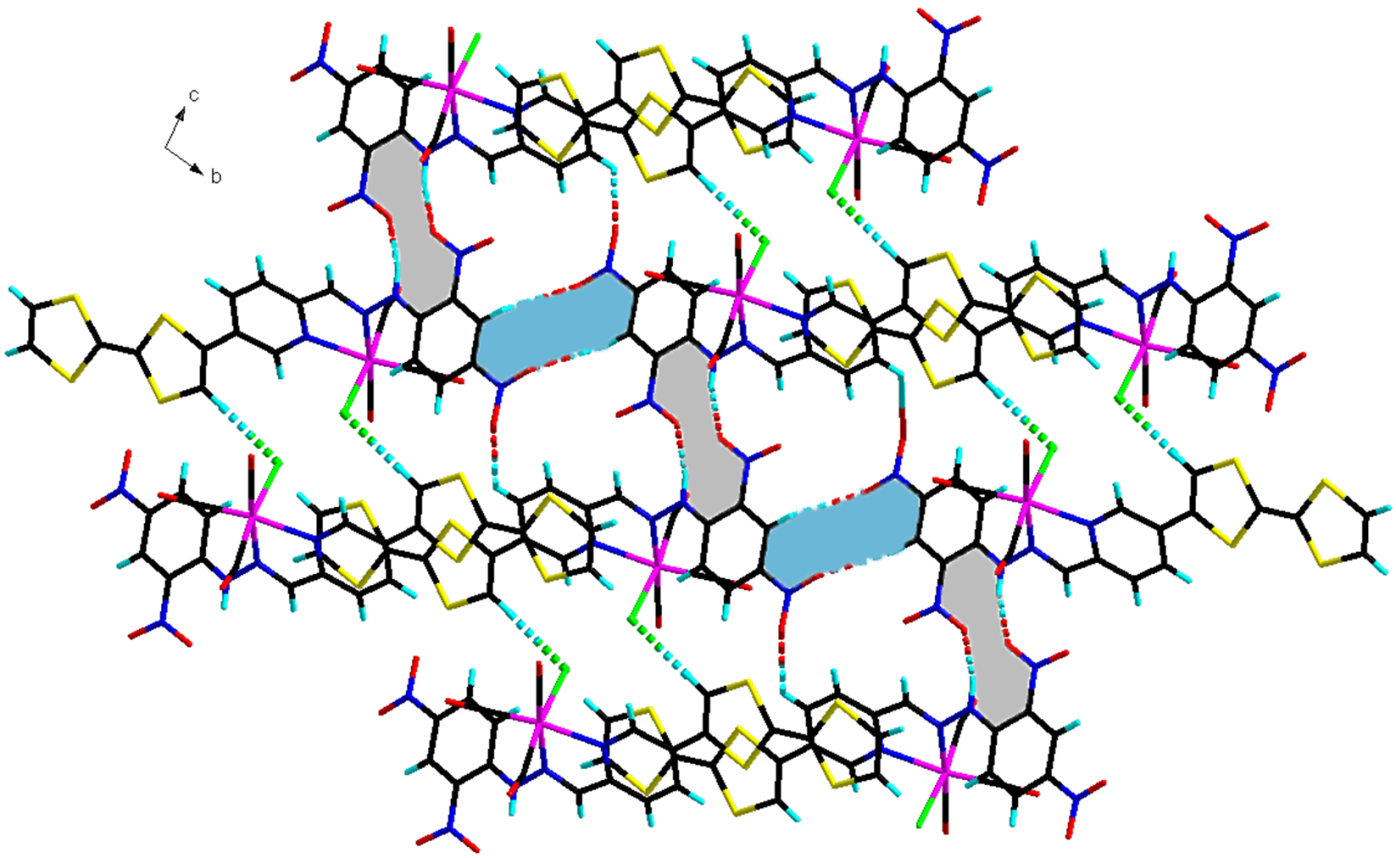
Layered structure of complex **3** viewed along the *a*-axis. The dimers are linked together through hydrogen bonding that form R_2_^2^(10) in blue filling and R_2_^2^(12) in grey filling cyclic motifs.

Adjacent dimers interact through hydrogen bonding interaction C–H···O (H···O 2.70(2) Å) formed between the NO_2_ group and an aromatic C–H that results in the establishment of R_2_^2^(10) cyclic motifs (blue filling in Figure 11) and N–H···O (H···O 2.32(2) Å) hydrogen bonds formed between the second NO_2_ group and N–H that form R_2_^2^(12) cyclic motifs (grey filling in Figure 11). This hydrogen bonding link therefore the molecules together into layers parallel with the *bc* crystallographic plane. The dimers of the resulting layers form a stack along *a*-axis through S···S contacts (d(S···S) being between 3.70(6) and 3.90(7) Å) resulting into a 3D supramolecular network.

The UV–visible absorption spectrum of the rhenium complex **3** recorded in a mixture of dichloromethane/acetonitrile (9:1, v/v) at room temperature (*c* 1.1 × 10^−4^ M) presents the same features as the free ligand **L2** with a red shift of the different absorption bands (Figure S10 in Supporting Information File 1). The ICT trasnsition suffers a bathochromic shift by about 100 nm as compared with the free ligand which indicates an increase of the electron acceptor effet of the ligand upon complexation with rhenium which acts as strong Lewis acid.

After complexation, the redox behavior of the TTF moiety is maintained. We note a positive shift of the two oxidation potentials in the case of complex **3** by about 90 mV and 100 mV (Figure S11 in Supporting Information File 1). This increase of the oxidation potential suggests that the rhenium fragment is acting as an electron acceptor by decreasing the electron density on the TTF unit. This behavior is in agreement with the electronic absorption experiments and confirms the strong electronic conjugation in ligand **L2**. The electrochemical behavior observed for **L2** and its corresponding rhenium complex **3** indicate that this compounds are valuable candidates for the electrochemical formation of air-stable radical cation crystalline salts [16].

## Conclusion

Two multifunctional ligands which associate an electron-donating TTF unit with an electron-accepting dinitrophenyl group as well as a coordinating pyridine azine moiety were successfully synthesized. Ligand **L2** exhibit a strong electronic conjugation between the donor and the acceptor resulting in the occurrence of an intramolecular charge transfer (ICT) band between the two fragments. Single crystals of ligand **L1** have been obtained and its crystal structure indicates the ligand is completely planar with the occurrence of a strong intramolecular as well as intermolecular hydrogen bonding. Inorganic anions titration experiments showed that the two ligands are suitable candidates for the sensing of fluoride anions. Metal cation-coordination experiments afforded the obtaining of a neutral electroactive rhenium(I) complex. The crystal structure of this complex indicates the formation of dimers that are connected through strong hydrogen bonding. The electrochemical behavior of both the ligands and the neutral rhenium(I) complex suggests that crystalline radical cation salts can be readily obtained upon chemical and/or electrochemical oxidation. The complexation abililty of the two novel electroactive ligands toward transition metal cations such as Cu(II), Fe(II), Co(II), etc is in progress.

## Experimental

### General information

NMR spectra were recorded on a Bruker Avance DRX 300 spectrometer operating at 300 MHz for ^1^H NMR and 75 MHz for ^13^C NMR. Chemical shifts are expressed in parts per million (ppm) downfield from external TMS. UV–visible spectra were recorded at room temperature in quartz cuvettes using Perkin Elmer spectrophotometer. Mass spectra were collected with Bruker Biflex-III TM. IR spectra were recorded on a Bruker vertex 70. Elemental (C, H and N) analyses were performed on a Thermo-Scientific Flash 2000 Organic Elemental Analyzer. Cyclic voltammetry (CV) experiments were performed in a three-electrode cell equipped with a platinum millielectrode as the working electrode , a platinum wire as a counter electrode and a silver wire Ag/Ag^+^ used as a reference electrode. The electrolytic media involved a 0.1 mol/L solution of (*n-*Bu_4_N)PF_6_ in dichloromethane/acetonitrile (9:1, v/v). Melting points were measured with a Melting Point Apparatus SMP3.

X-ray single-crystal diffraction data for complex **3** were collected at 180 K on an Agilent SuperNova diffractometer equipped with Atlas CCD detector and mirror monochromated micro-focus Cu K_α_ radiation (λ = 1.54184 Å). For ligand **L1**, crystal data were collected at 293 K on a Bruker KappaCCD diffractometer, equipped with a graphite monochromator utilizing MoKα radiation (λ = 0.71073Å). The two structures were solved by direct methods, expanded and refined on F^2^ by full matrix least-squares techniques using SHELX97 programs (G.M. Sheldrick, 1998). All non-H atoms were refined anisotropically and the H atoms were included in the calculation without refinement. Multiscan empirical absorption was corrected using the SADABS program (Bruker AXS area detector scaling and absorption correction, v2008/1, Sheldrick, G.M., (2008)) for ligand **L1** and using the CrysAlisPro program (CrysAlisPro, Agilent Technologies, V1.171.37.35g, 2014) for complex **3**. For ligand **L1**, the structure refinement showed disordered electron density which could not be reliably modeled and the program PLATON/SQUEEZE were used to remove the scattering contribution corresponding to dimethyl sulfoxide solvent from the intensity data. The assumed solvent composition (3 DMSO per asymmetric unit) was used in the calculation of the empirical formula, formula weight, density, linear absorption coefficient and F(000). For complex **3**, the largest difference peak and hole of 2.29 eÅ^−3^ observed is relatively high and it can be attributed to bad absorption correction. As the Gaussian absorption method does not improve the refinement, we have chosen the empirical absorption correction. This residual electronic density is located around the Re metal ion.

**6-([2,2’-Bi(1,3-dithiolylidene)]-4-yl)picolinaldehyde (1):** This compound was prepared as previously described [38]. Stannylated tetrathiafulvalene (0.50 g, 1.36 mmol) and 6-bromo-2-pyridinecarboxaldehyde (0.34 g, 1.36 mmol) were dissolved in toluene (20 mL) and [Pd(PPh_3_)_4_] (0.156 g, 0.135 mmol) was added. The reaction mixture was heated for 48 hours at 110 °C. After evaporation of the solvent under reduced pressure, the obtained residue was then passed over a silica gel column chromatography using a gradient of eluent (pentane/dichloromethane, 3:1, v/v). After solvent evaporation, a solid was obtained in 60% yield, (0.250 g, 0.809 mmol); mp 152 °C; ^1^H NMR (300 MHz, DMSO-*d*_6_) δ 9.95 (s, 1H), 8.19 (d, *J* = 7.21 Hz, 1H), 8.10 (t, *J* = 7.89 Hz, 1H), 7.88 (s, 1H), 7.85 (d, *J* = 7.50 Hz, 1H), 6.78 (s, 2H) ppm; ^13^C NMR (75 MHz, DMSO-*d*_6_) δ 193.3, 152.0, 150.9, 138.8, 136.7, 124.2, 122.3, 121.2, 120.7, 120.5, 112.3, 107.5 ppm; anal. calcd for C_12_H_7_NOS_4_: C, 46.58; H, 2.28; N, 4.53; found: C, 46.16; H, 2.22; N, 4.43; MALDI–TOF MS calcd: *m*/*z* = 309.5. found: *m*/*z* = 308.9 [M]^+^; HRMS (M): calcd for C_12_H_7_NOS_4_: 308.9410; found: 308.9413.

**5-([2,2’-Bi(1,3-dithiolylidene)]-4-yl)picolinaldehyde (2):** This compound was prepared by following the same procedure as for compound **1**. Yield (65%); mp 191 °C; ^1^H NMR (300 MHz, DMSO-*d*_6_) δ 9.98 (s, 1H), 8.97 (d, *J* = 1.65 Hz, 1H), 8.07 (dd, *J* = 6.42 Hz, *J* = 2.08 Hz, 1H), 7.96 (d, *J* = 8.30 Hz, 1H), 7.78 (s, 1H), 6.81 (s, 2H) ppm; ^13^C NMR (75 MHz, DMSO-*d*_6_) δ 193.2, 151.5, 147.4, 134.7, 131.8, 130.8, 122.6, 122.5, 120.7, 120.6, 114.2, 105.7 ppm; anal. calcd for C_12_H_7_NOS_4_: C, 46.58; H, 2.28; N, 4.53; found: C, 46.54; H, 2.20; N, 4.51; MALDI–TOF MS calcd: *m*/*z* = 309.5. found: *m*/*z* = 308.9 [M]^+^. HRMS (M): calcd for C_12_H_7_NOS_4_: 308.9410; found: 308.9404.

**2-([2,2’-Bi(1,3-dithiolylidene)]-4-yl)-6-((2,4-dinitrophenyl)hydrazono)methyl)pyridine (L1)**: 2,4-Dinitrophenylhydrazine (0.150 g, 0.757 mmol) and 6-([2,2’-bi(1,3-dithiolylidene)]-4-yl)picolinaldehyde (**1**, 0.234 g, 0.757 mmol) were dissolved in ethanol (20 mL) and three drops of acetic acid were added. The resulting solution was refluxed overnight. After cooling to room temperature, a dark precipitate was formed which was filtered and washed with ethanol then dried under vacuum to afford a dark powder of ligand **L1**, 75% (0.277 g, 0.567 mmol); mp 264 °C; ^1^H NMR (300 MHZ, DMSO-*d*_6_) δ 11.87 (s, 1H, -NH), 8.90 (d, *J* = 2.60 Hz, 1H), 8.78 (s, 1H), 8.43 (dd, *J* = 6.48 Hz, *J* = 2.70 Hz, 1H), 8.19 (d, *J* = 9.40 Hz, 1H), 7.98 (m, 3H), 7.78 (s, 1H), 6.78 (s, 2H) ppm; ^13^C NMR (75 MHz, DMSO-*d*_6_) δ 160.8, 154.6, 147.3, 144.7, 141.6, 140.6, 138.4, 134.2, 134.1, 131.0, 130.2, 129.2, 123.3, 120.5, 117.5 ppm; selected IR bands (cm^−1^): 1614, 1499, 1333; anal. calcd for C_18_H_11_N_5_O_4_S_4_: C, 44.15; H, 2.26; N, 14.30, found: C, 43.54; H, 2.22; N, 13.66; MALDI–TOF MS calcd: *m*/*z* = 489.6. found: *m*/*z* = 489.1 [M]^+^; HRMS (M): calcd for C_18_H_11_N_5_O_4_S_4_: 488.9694; found: 488.9687.

**5-([2,2’-bi(1,3-dithiolylidene)]-4-yl)-2-((2,4-dinitrophenyl)hydrazono)methyl)pyridine (L2)**: This ligand was prepared by following the same procedure used for **L1**. Yield: 63%; mp 257 °C; ^1^H NMR (300 MHz, DMSO-*d*_6_) δ 11.88 (s, 1H, -NH), 8.88 (d, *J* = 2.64 Hz, 1H), 8.75 (d, *J* = 2.34 Hz , 1H), 8.72 (s, 1H), 8.41 (dd, *J* = 6.96 Hz, *J* = 2.63 Hz, 1H), 8.17 (d, *J* = 9.63 Hz, 1H), 8.11 (d, *J* = 8.13 Hz , 1H), 7.96 (dd, *J* = 5.99 Hz, *J* = 2.40 Hz, 1H), 7.58 (s, 1H), 6.79 (s, 2H) ppm; ^13^C NMR (75 MHz, DMSO-*d*_6_) δ 152.6, 148.6, 147, 144.7, 138.3, 134.2, 131.3, 130.8, 130.3, 128.6, 123.3, 121.1, 120.7, 117.7, 113.6, 106.3 ppm; selected IR bands (cm^−1^): 1612, 1508, 1325; anal. calcd for C_18_H_11_N_5_O_4_S_4_: C, 44.15; H, 2.26; N, 14.30; S, 26.19, found: C, 43.69; H, 2.16; N, 13.98; S, 26.01; MALDI–TOF MS calcd: *m*/*z* = 489.6. found: *m*/*z* = 489.0 [M]^+^; HRMS (M): calcd for C_18_H_11_N_5_O_4_S_4_: 488.9694; found: 488.9703.

**Rhenium(I) complex [ReL2(CO)_3_Cl] 3:** To a solution of ligand **L2** (0.025 g, 0.051 mmol) in a mixture of toluene and dichloromethane (3:1, v/v) solution was added [Re(CO)_5_Cl] (0.027 g, 0.076 mmol). The mixture was refluxed for 6 hours under nitrogen atmosphere. After cooling the resulting mixture to room temperature, the solvent was removed by a rotary evaporator. The brown residue was extracted with dichloromethane and recrystallized from acetone/hexane solvent mixture to yield complex **3** as black crystals with 81% yield (0.033 g, 0.041 mmol); mp > 360 °C; ^1^H NMR (300 MHz, DMSO-*d*_6_) δ 12.35 (s, 1H), 9.36 (m, 1H), 9.02 (s, 1H), 8.91 (s, 1H), 8.32 (m, 3H), 8.0 (s, 1H), 7.84 (m, 1H), 6.83 (s, 2H) ppm; ^13^C NMR (75 MHz, DMSO-*d*_6_) δ 197.6, 196.8, 193.0, 152.5, 148.5, 147.6, 144.7, 138.2, 136.4, 134.1, 131.2, 130.8, 130.2, 129.2, 128.5, 123.3, 121.0, 120.7, 117.7, 113.6, 106.3 ppm; selected IR bands (cm^−1^): 2018, 1868, 1614, 1497, 1333; MALDI–TOF MS calcd: *m*/*z* = 795.3. found: *m*/*z* = 795.2 [M]^+^; HRMS (M): calcd for C_21_H_11_O_7_N_5_S_4_ReCl: 794.8787; found: 794.8781.

## Supporting Information

File 1 Additional analytical data.
