# Supplementary material for: Tetrathiafulvalene-based azine ligands for anion and metal cation coordination
Source: Beilstein J Org Chem. 2015 Aug 7;11:1379–91. doi: 10.3762/bjoc.11.149 (PMC4578403; doi:10.3762/bjoc.11.149)
Supplement: File 1 — Additional analytical data. [file Beilstein_J_Org_Chem-11-1379-s001.pdf]

Supporting Information

for

Tetrathiafulvalene-based azine ligands for anion and metal cation coordination

Awatef Ayadi[1,2], Aziz El Alamy[3], Olivier Alévêque[1], Magali Allain[1], Nabil Zouari[2], Mohammed Bouachrine[3] and Abdelkrim El-Ghayoury[1]*

Address: [1]Laboratoire MOLTECH Anjou, Université d'Angers, UFR Sciences, UMR 6200, CNRS, Bât. K, 2 Bd. Lavoisier, 49045 Angers Cedex, France, [2]Laboratoire *de* Physico-chimie de l'état solide, Université de Sfax, Route de Soukra; Km 4; BP: 802, 3038, Sfax, Tunisia and [3]MEM, High School of Technology (ESTM), University, Moulay Ismail, Meknès, Morocco

E-mail: Abdelkrim El-Ghayoury* - abdelkrim.elghayoury@univ-angers.fr

*Corresponding author

Additional analytical data

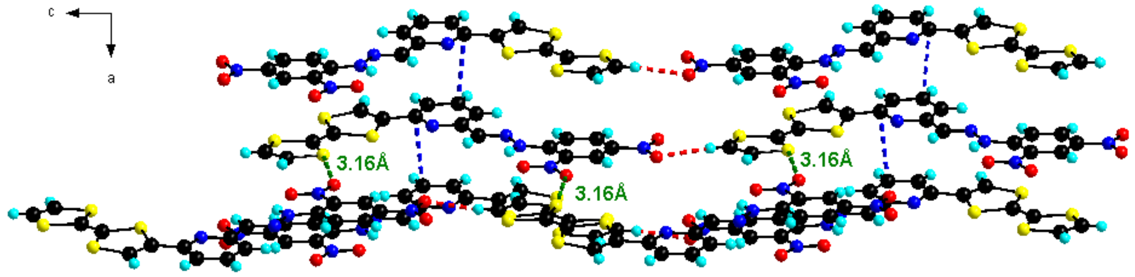

Figure S1: Partial crystal packing of ligand **L1** with columns of stacked head to tail molecules that are connected laterally through S···O heteroatom contacts.

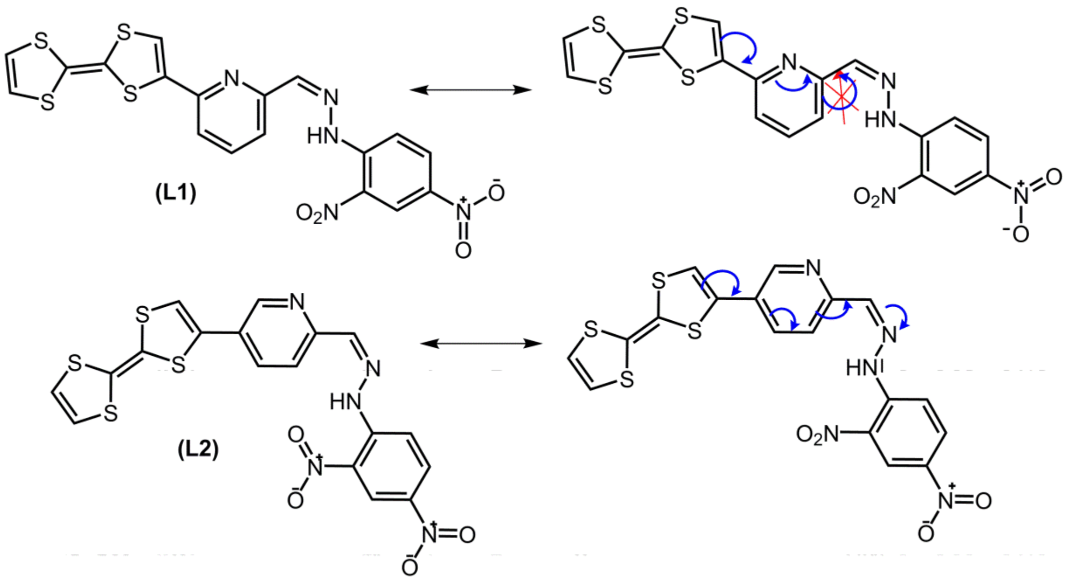

Figure S2: Electronic delocalization scheme for ligands **L1** and **L2**.

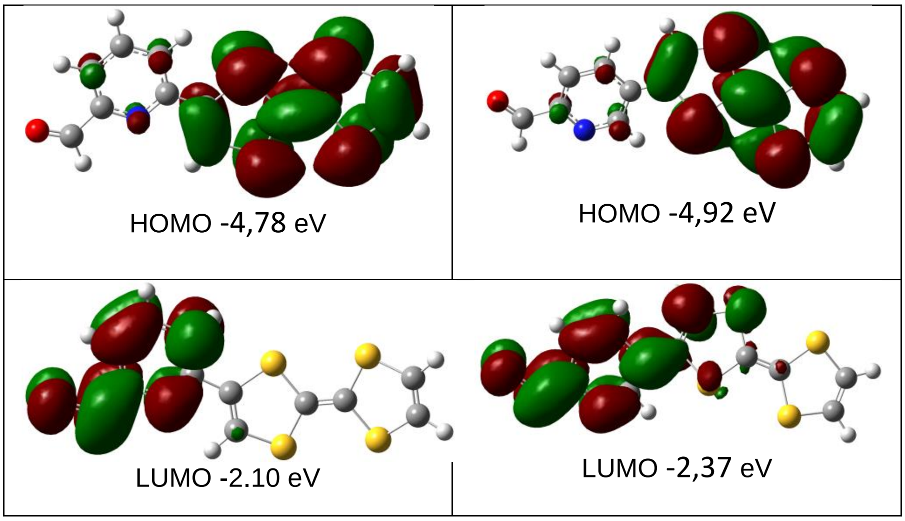

Figure S3: HOMO-LUMO Frontier orbitals representation for compounds **1** and **2**.

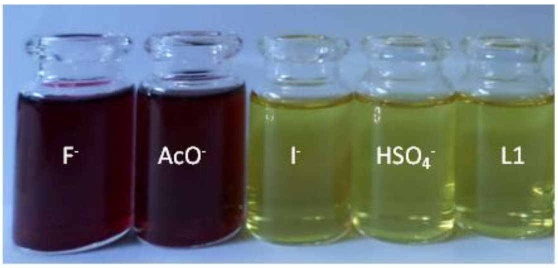

Figure S4: Color change of ligand **L1** upon addition of inorganic anions.

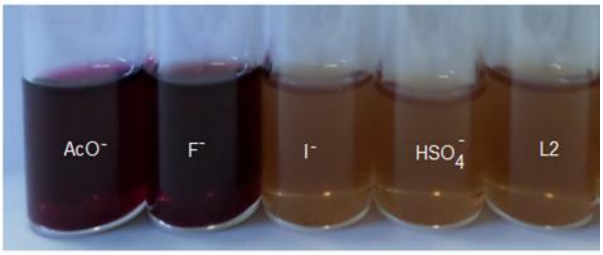

Figure S5: Color change of ligand **L2** upon addition of inorganic anions.

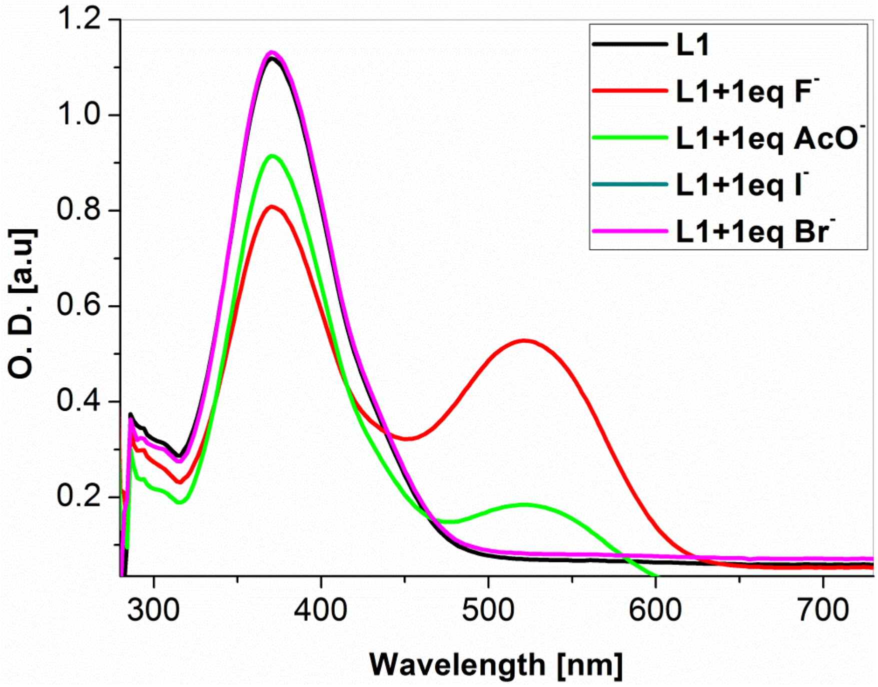

Figure S6: UV-visible absorption spectra of **L1** upon addition of 1 equiv of anion.

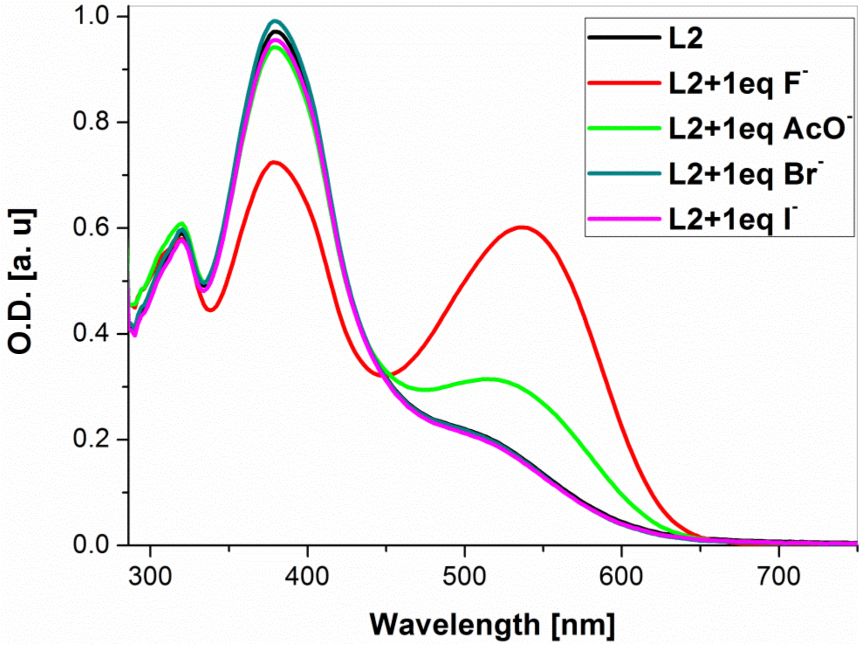

Figure S7: UV-visible absorption spectra of **L2** upon addition of 1 equiv of anion.

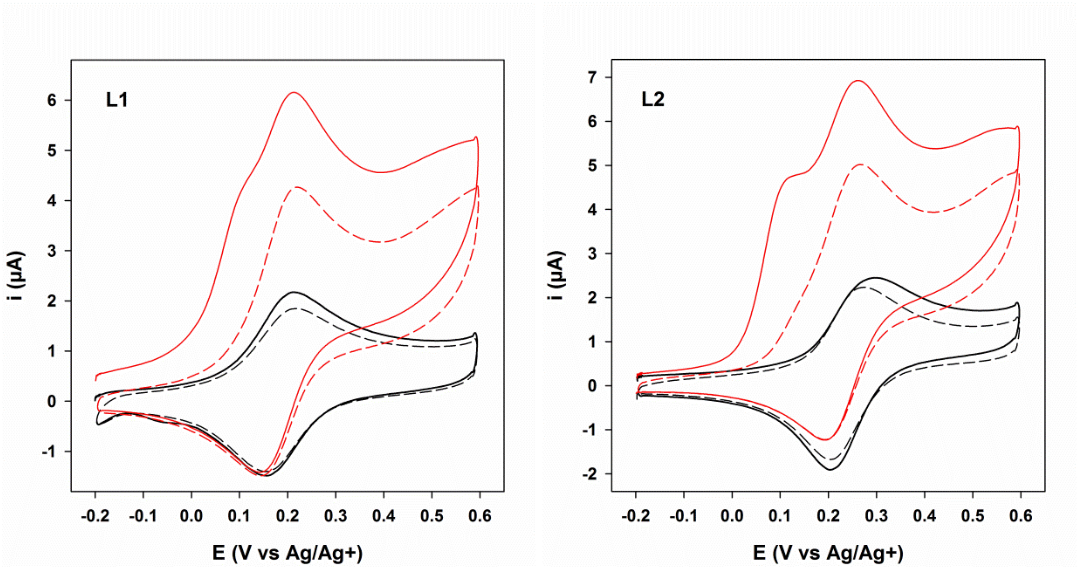

Figure S8: Cyclic voltammograms of ligands **L1** (left) and **L2** (right) (2×10^{-5} M) without (black, bold lines first cycle and dashed lines for second cycle) and with (red, bold lines first cycle and dashed lines for second cycle) 2 equiv of F^-. CVs recorded at 100 mV·s^{-1} on a glassy carbon electrode in CH_2Cl_2/CH_3CN (9/1, v/v) with *n*-Bu_4NPF_6 (0.1 M).

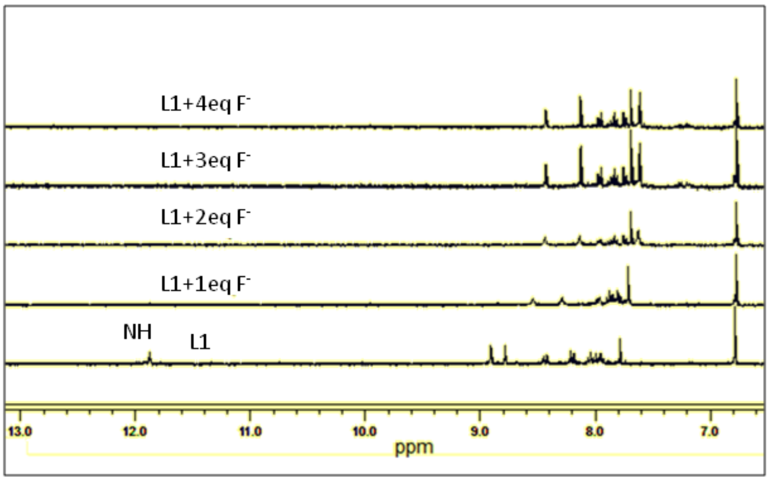

Figure S9: ^{1}H NMR spectra of ligand **L1** (4×10^{-3} M in DMSO-d_6) upon addition of successive aliquots of TBAF (DMSO-d_6).

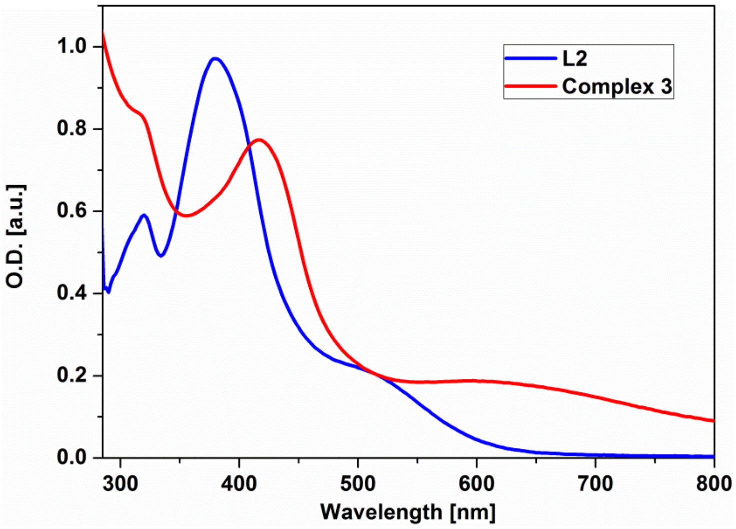

Figure S10: UV–visible absorption spectra of complex **3** (*c* 1.1 × 10^{-4} M in dichloromethane/acetonitrile, 9/1, (v/v)), room temperature. Ligand **L2** is added for comparison.

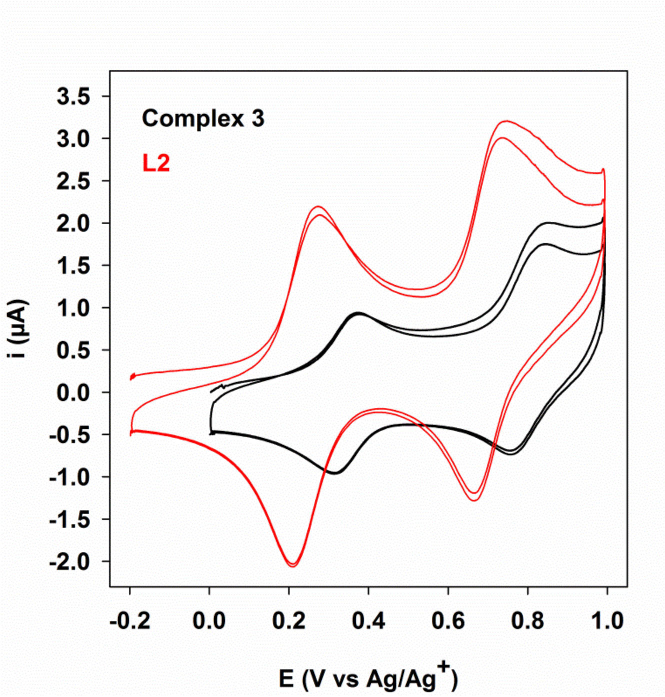

Figure S11: Cyclic voltammograms of **L2** (red, added for comparison) and complex **3** (black) (1.1 × 10^{-4} M) at 100 mVs^{-1} on a glassy carbon electrode in CH_2Cl_2/CH_3CN (9/1, v/v) with *n*-Bu_4NPF_6 (0.1 M).
